# The Royal College of Ophthalmologists' National Ophthalmology Database study of cataract surgery: report 1, visual outcomes and complications

**DOI:** 10.1038/eye.2015.3

**Published:** 2015-02-13

**Authors:** A C Day, P H J Donachie, J M Sparrow, R L Johnston

**Affiliations:** 1 The NIHR Biomedical Research Centre at Moorfields Eye Hospital NHS Foundation Trust and UCL Institute of Ophthalmology, London, UK; 2 The Royal College of Ophthalmologists' National Ophthalmology Database, London, UK; 3 Gloucestershire Hospitals NHS Foundation Trust, Cheltenham, UK; 4 Department of Ophthalmology, Bristol Eye Hospital, Bristol, UK

## Abstract

***Aims*:**

To describe the outcomes of cataract surgery in the United Kingdom.

***Methods*:**

Anonymised data on 180 114 eyes from 127 685 patients undergoing cataract surgery between August 2006 and November 2010 were collected prospectively from 28 sites. Outcome measures included intraoperative and postoperative complication rates, and preoperative and postoperative visual acuities.

***Results*:**

Median age at first eye surgery was 77.1 years, 36.9% cases had ocular co-pathology and 41.0% patients underwent cataract surgery on both eyes. Preoperative visual acuity was 0.30 logMAR or better in 32.0% first eyes and 47.7% second eyes. Postoperative best-measured visual acuity was 0.00 and 0.30 logMAR or better in 50.8 and 94.6% eyes without ocular co-pathology, and 32.5 and 79.9% in eyes with co-pathology. For eyes without co-pathology, postoperative uncorrected distance visual acuity was 0.00 and 0.30 logMAR or better in 27.3 and 80.9% eyes. Posterior capsule rupture or vitreous loss or both occurred in 1.95% cases, and was associated with a 42 times higher risk of retinal detachment surgery within 3 months and an eight times higher risk of endophthalmitis.

***Conclusion*:**

These results provide updated data for the benchmarking of cataract surgery. Visual outcomes, and the rate of posterior capsule rupture or vitreous loss or both appear stable over the past decade.

## Introduction

Cataract is the leading cause of blindness in the world^1^ and cataract surgery is the most commonly performed operation by the UK National Health Service (NHS) with around 330 000 cataract operations performed per year in England. Robust data are critical for the benchmarking of surgical outcomes, as a quality improvement tool, and for commissioners and patients to make informed choices.

The Royal College of Ophthalmologists' National Ophthalmology Database (RCOphth NOD) was established to provide national audit and research data, and to provide an evidence base for revalidation standards allowing Ophthalmologists to compare their surgical outcomes with those of their anonymised peers. The RCOphth NOD is the formalised successor to the *ad hoc* collaboration that resulted in the Cataract National Dataset publications.^2, 3, 4, 5, 6^ The RCOphth NOD covers a range of conditions and operations^7, 8, 9, 10^ and collates pseudoanonymized data collected during routine clinical care using electronic medical record systems (EMRs).

## Materials and methods

Data were extracted from 31 UK NHS Trusts of which 28 had recorded data for cataract surgery, as detailed in the acknowledgements section. All cataract operations were performed between August 2006 and November 2010 and this time frame was specifically chosen to exclude data used in previous analyses of cataract surgery outcomes in the Cataract National Dataset publications.^2, 3, 4, 5, 6^ All data were recorded using a single EMR system (Medisoft Ophthalmology, Medisoft Limited, Leeds, UK). The lead clinician and Caldicott Guardian (responsible nominee for data protection) at each NHS Trust gave written approval for anonymised data extraction. Anonymized database analyses of this type do not require ethical permission as they are viewed as audit or service evaluation (see http://www.hra.nhs.uk/research-community/before-you-apply/determine-whether-your-study-is-research/) and this was confirmed by a Research Ethics Committee. This study was conducted in accordance with the declaration of Helsinki, and the UK's Data Protection Act.

### Case definitions

Eligible cataract operations were those performed on patients aged 18 years or older using phacoemulsification and where the primary intention was cataract surgery, not combined ‘cataract+other' operations where the cataract component may not have been the primary reason for surgery. Surgeon grades were categorised as: Consultant surgeons, independent non-consultant surgeons (staff grades, associate specialists and trust doctors) and trainee surgeons (Foundation Year doctors, Specialist Trainees, Senior House Officers, Specialist Registrars and Fellows.) Owing to progression through training an individual surgeon can have data recorded at more than one grade. Ocular co-pathology was a compulsory question with responses of none, a list of common categories and a free text field for rare options.

### Intraoperative complications

In all centres the EMR software mandated the collection of the presence or absence of surgical complications. If the surgeon indicated that a complication occurred, then they had to select from a pre-populated list of complications specific to that operation, or select ‘other' and record the complication using free text.

‘Posterior Capsular Rupture or vitreous loss or both' (abbreviated as PCR) was defined as unintentional communication with the posterior segment from the occurrence of any of the following intraoperative complications: PCR with or without vitreous loss, zonule rupture with vitreous loss, vitreous loss, vitreous to the section at the end of surgery, IOL into the vitreous or nuclear/epinuclear fragment into the vitreous or the performing of any of an automated anterior vitrectomy, ‘sponge and scissors vitrectomy,' secondary IOL or scleral fixed IOL during surgery; or if either of vitreous to the section or anterior chamber were recorded within 2 months of cataract surgery, or an operation for a dropped nucleus was recorded within 3 months of cataract surgery.

### Postoperative complications: retinal detachment and endophthalmitis

Owing to varying availability of postoperative complication data, the results for post cataract retinal detachment surgery and endophthalmitis treatment were confined to centres where this could be cross-checked with other RCOphth NOD treatment data. Data on retinal detachment surgery were available from 15 centres providing 139 537 (77.5%) of all cataract operations and results are reported for within 3 months, 6 months and 1 year of cataract surgery. For endophthalmitis data from 19 centres providing 145 868 (81.0%) cataract operations were available and results are reported within 3 months of cataract surgery.

### Visual acuity

Preoperative visual acuity data was defined as the better value of uncorrected distance visual acuity (UDVA) or corrected distance visual acuity (CDVA). Eligible VA measurements were recorded within 3 months prior to cataract surgery where the VA measurement closest to the date of cataract surgery was used in the analysis.

Postoperative ‘best-measured' visual acuity was defined as the best CDVA measurement within 2 weeks and 4 months of cataract surgery when present and the best measurement of UDVA or pinhole VA within the time period when CDVA was absent.

For postoperative UDVA only analysis, data was used for eyes where the predicted postoperative refraction was ±0.5 dioptres spherical equivalent.

Visual acuity values were recorded as Snellen or logMAR, and Snellen values were converted to logMAR for analyses. LogMAR values corresponding to count fingers (CF), hand movements (HM), perception of light (PL) and no PL (NPL) were substituted with 2.10, 2.40, 2.70 and 3.00 logMAR, respectively, in keeping with previous publications from this group.

Significant visual loss was defined as visual acuity deteriorating by ≥0.30 logMAR units (doubling or worse of the visual angle)^4^ between preoperative and postoperative measures.

Fishers exact test or Pearson's χ^2^ test were used as appropriate and all analysis was conducted using STATA version 11 (StataCorp, College Station, TX, USA) except for the calculation of confidence intervals which were calculated using Confidence Interval Analysis version 2.1.2.^11^

## Results

There were 180 114 cataract operations performed on 127 685 patients eligible for analysis. These were performed by 995 surgeons at 27 NHS Trusts in England and 1 in Scotland. Median patient age at first eye cataract surgery was 77.1 years (IQR: 69.7–82.8); where 51 838 (40.6%) patients were male, 75 465 (59.1%) female and 382 (0.3%) gender not specified.

In 67.3% cases the operation was on the patient's first eye to undergo cataract surgery. For those who had surgery on both eyes (41.0% of all patients), the median time between operations was 3.7 months (IQR: 2.4–6.6, range: 0–114.6 months). 436 (0.3%) patients underwent immediate sequential bilateral surgery performed by 120 surgeons.

### Grade of surgeon

Of the 995 surgeons, 285 were Consultant surgeons, 451 independent non-consultant surgeons and 325 trainee surgeons. Sixty-six surgeons performed surgery while at more than one grade of surgeon.

### Surgical details

Local anaesthesia was used for 96.2% operations, general anaesthesia for 3.5% operations and not known for 0.3% operations. Pupil size at the time of surgery was considered to be ‘small,' ‘medium,' or ‘large' in 3.0, 10.7, and 86.2% eyes, respectively, and was not recorded for 0.1% of eyes. The intraocular lens was placed in the capsular bag in 98.6% eyes, in the sulcus or partly in the bag for 1.0% eyes, and in the anterior chamber for 0.1% eyes. In 0.2% eyes, no intraocular lens was placed (aphakic) and in 0.2% of the intraocular lens position was not recorded.

### Ocular co-pathology

Ocular co-pathology was present in 66,504 (36.9%) cases, with age-related macular degeneration (10.0%), glaucoma (8.0%), and diabetic retinopathy (4.7%) being the most frequently recorded (Table 1). Consultant surgeons performed a higher proportion of their operations on eyes with an ocular co-pathology (*P*=0.000).

### Intraoperative complications

Overall 4.2% (95% CI: 4.1–4.3%) cases had an intraoperative complication, the most common being PCR (1.95%, 95% CI: 1.89–2.02% Table 2). The rate of PCR was 1.63% in eyes without co-pathology (1847/113 610) and 2.51% (1667/66 504) in those eyes with a co-pathology. The presence of at least one ocular co-pathology was associated with any intraoperative complication (OR 1.53, 95% CI: 1.46–1.61, *P*=0.000) and PCR (OR 1.56, 95% CI 1.46–1.66, *P*=0.000).

**Figure 1:**
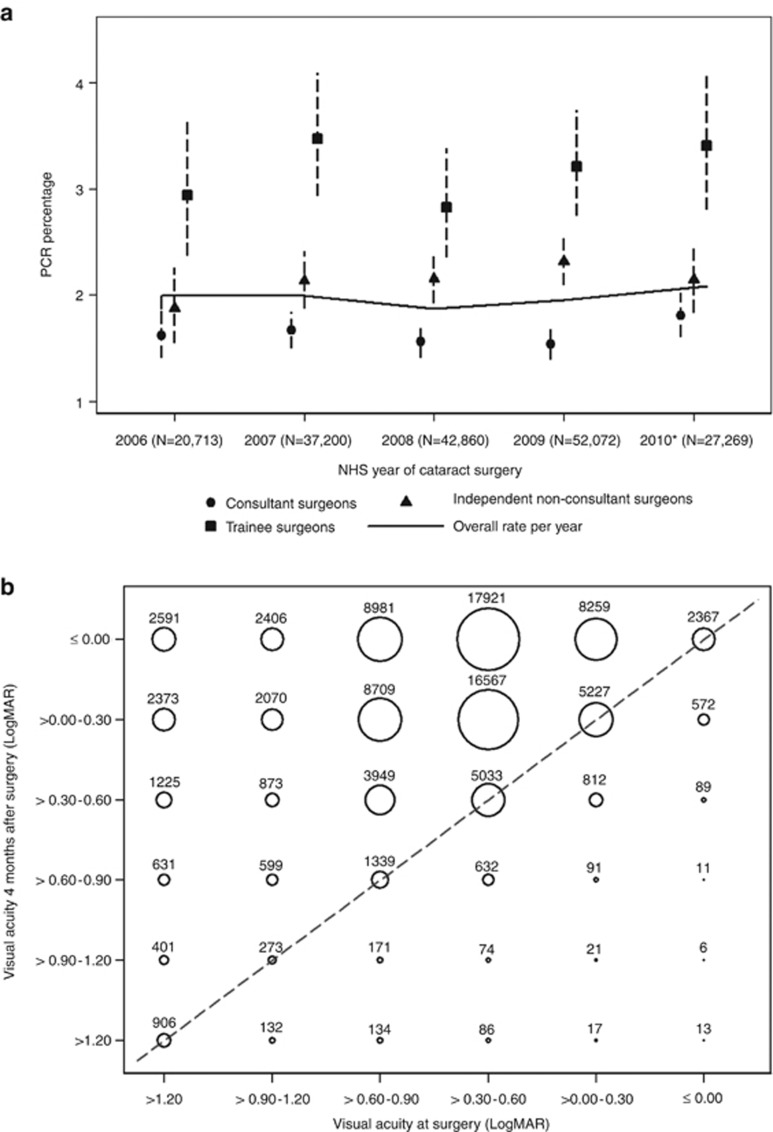
(a) Posterior capsular rupture and/or vitreous loss (PCR) rates by grade of surgeon and NHS year. The dashed lines represent 95% confidence intervals and the solid line joins the yearly PCR rates for all surgeons. The NHS year runs from 1 April to 31 March. The 2010 NHS year is not complete; data is displayed up to 30 November 2010. (b) Bubble plot of visual acuities for all eyes. The size of each circle is proportional to the total number of observations and the labels are the number of eyes. Of the 95 561 eyes 77 555 (81.2%) were in a higher VA category (above the dotted line), 15 145 (15.8%) remained in the same category (on the dotted line) and 2861 (3.0%) were in a lower VA category 4 months after cataract surgery compared with their presenting VA measurement.

Intraoperative complication rates were lower for consultant surgeons than other surgeon grades (*P*=0.000), see Table 2. Over the study period, the rate of PCR by surgical grade and the overall rate appears stable, see Figure 1a.

### Postoperative complications: retinal detachment and endophthalmitis

The rate of retinal detachment surgery within 3 months of cataract surgery was 0.03% (45/139 537 cases, 95% CI: 0.02%–0.04%), and this was ∼42 times higher (OR 41.66, 95% CI: 23.09–75.17, *P*=0.000) in eyes that had PCR than those without. Within 6 months of cataract surgery 73 eyes and within 1 year 108 eyes had surgery for retinal detachment. For both time points, higher proportions of eyes that had PCR underwent retinal detachment surgery, OR 23.98 (95% CI: 14.60–39.37) and 18.28 (95% CI: 11.86–28.17) respectively.

The rate of endophthalmitis within 3 months of cataract surgery was 0.03% (43/145,868 cases, 95% CI: 0.02–0.04%). The rate of endophthalmitis was approximately eight times higher (OR 7.94, 95% CI: 3.35–18.83, *P*=0.000) in cases with PCR than those without.

### Visual acuity

Preoperative visual acuity data were available for 147 962 (82.1%) eyes, of which 100 473 (67.9%) were CDVA measurements, 43 537 (29.4%) were UDVA measurements and in 3952 (2.7%) cases the CDVA equalled the UDVA. The median VA was 0.50 logMAR (IQR: 0.30–0.80), and mean 0.63 logMAR (including 6905 (4.7%) eyes with CF, 2973 (2.0%) with HM, 819 (0.6%) with PL, and 50 (<0.1%) with NPL).

For all eyes, 3.2, 4.6, and 36.2% had a preoperative visual acuity of 0.00, 0.18, or 0.30 logMAR or better. For those without ocular co-pathology the corresponding values were 3.7, 5.2, 40.7% respectively. Preoperative visual acuity was 0.00, 0.18, or 0.30 logMAR or better in 2.5, 3.5, and 32.0% of first eyes (*n*=108 086); and 5.1, 7.4, and 47.7% of second eyes (*n*=39 876).

Of those with preoperative visual acuity data, 95 561 (64.6%) had postoperative visual acuity data between 2 weeks and 4 months after surgery (median 61 days). Of the 95 561 postoperative VA measurements, 64 421 (67.4%) were CDVA measurements, 20 617 (21.6%) were UDVA measurements and 10 523 (11.0%) were pinhole measurements. The median postoperative best-measured visual acuity was 0.10 logMAR (IQR: 0.00–0.20) and the mean was 0.16 logMAR (SD: 0.30; including 576 (0.6%) eyes with CF, 232 (0.2%) with HM, 54 (<0.1%) with PL, and 6 (<0.1%) with NPL). For the 62 848 eyes with no ocular co-pathology, the median postoperative visual acuity was 0.00 logMAR (IQR: 0.00–0.20). The mean and standard deviation were 0.10 and 0.21 logMAR, respectively.

Table 3 shows the breakdown of postoperative visual acuity values. Figure 1b shows a bubble plot comparing preoperative and postoperative visual acuities. Postoperative UDVA for those with a predicted postoperative refraction of ±0.5 dioptres were available for 63 669 eyes (43 577 with no ocular co-pathology). The median postoperative UDVA was 0.20 logMAR (mean=0.27; SD=0.32) for all eyes and 0.20 logMAR (mean=0.22; SD-0.25) for those without ocular co-pathology.

### Visual acuity loss

Significant visual loss occurred in 1455 (1.5%) eyes, Table 4. A higher proportion of eyes with an ocular co-pathology, any intraoperative complication or PCR had visual loss (all *P*<0.001). The rate of visual loss did not differ across surgeons grade, 1.5% for consultant surgeons, 1.5% for independent non-consultant surgeons, and 1.7% for trainee surgeons (*P*=0.184).

## Discussion

This study provides pragmatic data on current cataract surgery outcomes using data collated from a large multi-centre database over 4 years. Overall, we found the median visual acuity improved from 0.50 before surgery to 0.10 logMAR after surgery. Comparison with the Cataract National Dataset analysis reporting on outcomes between 2001 and 2006 in the UK, suggests the rate of PCR and proportions achieving good postoperative visual acuities have stabilised.^2^

Over the past 20 years there has been a dramatic change in practice to operating on eyes with cataract at lower levels of visual impairment. The reasons for this are multifactorial and include better surgical techniques and outcomes, patients' visual expectations and a need for higher visual function to maintain independence, including that for driving.^12^ In 1990, 8.3% cases in England had a preoperative visual acuity of 0.30 logMAR or better,^13^ in 1997 this had increased to 31%,^14^ and in Cataract National Dataset analysis for 2001–2006 was 43%.^2^ In this series, 36% had a preoperative visual acuity of 0.30 logMAR or better. We found 32% first eyes and 48% second eyes had a preoperative visual acuity of 0.30 logMAR or better compared with 35 and 55%, respectively in the Cataract National Dataset papers.^2^ Thus suggesting there is no longer a trend towards performing cataract surgery at lower levels of visual impairment in the UK, and if anything this may have reversed slightly. This may be owing to cataract surgery rationing in recent years.^15^

The analyses of postoperative visual acuity showed 44 and 89% all eyes achieved 0.00 and 0.30 logMAR or better. These results are similar to those reported in the Cataract National Dataset paper where 46 and 91% were 0.00 logMAR and 0.30 logMAR or better, however immediate comparison is complicated by differing proportions of those with ocular co-pathology (37% RCOphth NOD *vs* 29% in the Cataract National Dataset). Part of this may be explained by a change in the EMR definition of co-pathology from conditions likely to be a cause of a guarded visual prognosis in the years contributing to Cataract National Dataset publications, to presence or absence of co-pathology in the years covered by this analysis. For those without ocular co-pathology, we found 50 and 94% were 0.00 and 0.30 logMAR or better, respectively compared with 51 and 95% in the Cataract National Dataset papers. We used slightly different definitions for pre and postoperative visual acuity measures than those in the Cataract National Dataset (we did not consider pinhole visual acuity as a proxy for missing preoperative visual acuity data, and used the better of UDVA and pinhole for absent postoperative CDVA data, rather than the best of UDVA, CDVA, or pinhole).^2^ Repeat analyses using the earlier definitions made no clinically meaningful difference to our results. In comparison with the outcomes from the recent European Registry of Quality Outcomes for Cataract and Refractive Surgery (EUREQUO) sub-analysis^16^ on 274 000 eyes without ocular co-pathology, our results appear slightly less favourable with EUREQUO reporting 70 and 98% had a postoperative CDVA of 0.00 and 0.30 logMAR or better. However, these are CDVAs only, so each case has undergone a formal refraction.

Cataract surgery does not always result in an improvement in visual acuity or patient satisfaction with visual function. The EUREQUO analysis reported that for those with a preoperative visual acuity of 0.00, 0.10, and 0.20 logMAR or better, 11.9, 2.3, and 1.4% cases had worse CDVA following surgery.^16^ For those with 0.00 logMAR preoperatively with glaucoma, macular degeneration, and diabetic retinopathy, 22, 22 and 28%, respectively, had worse visual acuity after surgery. In our analyses using a strict definition for vision loss of ≥0.30 logMAR units,^4^ we found for eyes without ocular co-pathology with preoperative visual acuities of 0.00, 0.18, and 0.30 logMAR or better, respectively, that 5.7, 4.6, and 2.4% had visual loss.

The PCR rate in our series was 1.95% and as seen in figure 1a, the value remained stable over the 4-year study period. The PCR rate is almost identical to that reported in the previous Cataract National Dataset analysis covering 2001–2006.^2^ Data from the Swedish National Cataract Register (NCR) on 602 553 operations performed between 2002 and 2009 reported an overall rate of a capsule complication (similar to our definition of PCR) of 2.1%,^17^ with a consistent decline until 2006 and stabilisation thereafter with rates of 1.6–1.9%.

A strength of this study is that the data were non-selective, pooled, and anonymized, so they may be more generalisable than data obtained from randomized controlled trials, and less subject to publication bias than single-centre case series.^18^ The 28 sites providing data for these analyses represent ∼15% of the annual number of cataract surgeries in England and Scotland as although there is encouragement to collect data to nationally agreed standards, each NHS Trust is independent resulting in variable adoption of EMR systems. All data used in these analyses were from a single EMR platform (Medisoft Ophthalmology) and were from Trusts that have agreed to contribute data to the NOD and were able to provide this. The recording of intraoperative complications is mandatory on the EMR and accuracy depends on surgeons recording their complications faithfully. It is not possible to confirm how reliably a surgeon records these, and surgeons may have a natural reluctance to record complications even though their results were anonymized. Data on possible under-reporting rates may be partly inferred by the <0.1% operations that had an unplanned anterior vitrectomy without PCR having been reported to have occurred. Another limitation of our study is incompleteness of particularly postoperative data, with for example, only 53.1% of eyes being eligible for the postoperative visual acuity analyses.

The finding of an eight times higher risk of endophthalmitis if PCR occurred is consistent with data from a recent meta-analysis that found PCR was associated with a six times higher risk of endophthalmitis,^19^ and was the intraoperative factor with most contribution to endophthalmitis risk.^19^ Although the association of PCR with a higher risk of retinal detachment is well known, our findings of 42, 24, and 18 times higher likelihoods of retinal detachment surgery within 3, 6, and 12 months, respectively, of cataract surgery if PCR occurred are higher than previously reported, but do suggest the difference in risk decreases with time. Our results are comparable with the Swedish NCR where a 15 times higher odds of retinal detachment surgery within 3 years was found if a capsule complication occurred.^20^ The rates for retinal detachment and endophthalmitis after cataract surgery (both 0.03%), must be interpreted with caution as they are likely to represent minimum values owing to the possibility that cases with these complications may have been treated at other NHS Trusts not contributing to this data set.

Overall, this analysis reports pragmatic data from a national database of cataract surgery outcomes allowing benchmarking of surgical units and individual surgeons, and gives an overview of the results that may be anticipated in the UK NHS. Our findings suggest that the trend of operating at lower levels of visual impairment has stopped or even reversed, and that visual outcomes and surgical complication rates have been stable over the past decade.


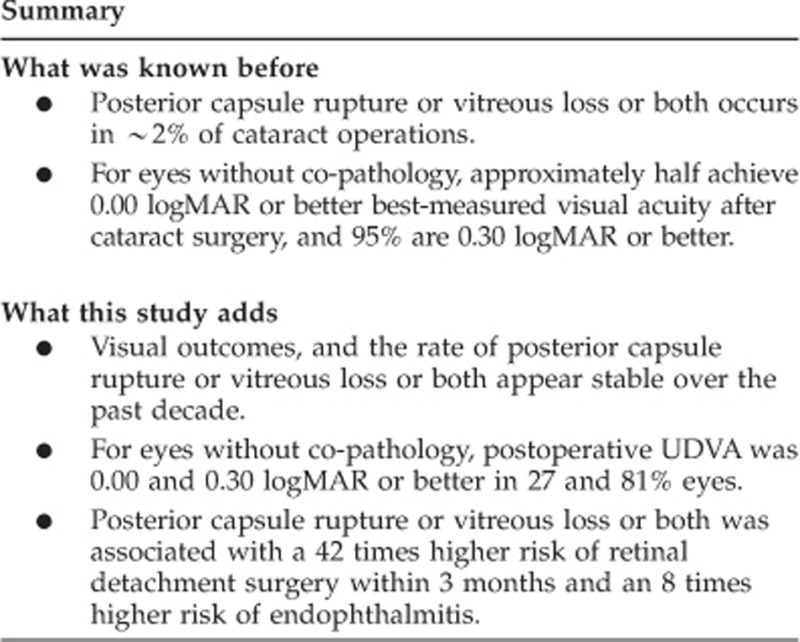


## Figures and Tables

**Table 1:** Ocular co-pathology in the operated eye by grade of operating surgeon

*Ocular co-pathology,* n *(column %)*	*Consultant surgeon*	*Independent non-consultant surgeon*	*Trainee surgeon*	*Total*
Number of eyes	105 116	56 359	18 639	180 114
Number of eyes without ocular co-pathology	62 986 (59.9)	37 693 (66.9)	12 931 (69.4)	113 610 (63.1)
Number of eyes with an ocular co-pathology	42 130 (40.1)	18 666 (33.1)	5708 (30.6)	66 504 (36.9)
1 co-pathology per eye	34 693 (33.0)	16 244 (28.8)	4971 (27.1)	55 908 (31.0)
2 co-pathologies per eye	6333 (6.0)	2118 (3.8)	646 (3.5)	9097 (5.1)
3 co-pathologies per eye	933 (0.9)	261 (0.5)	80 (0.4)	1274 (0.7)
≥4 co-pathologies per eye	171 (0.2)	43 (<0.1)	11 (<0.1)	225 (0.1)
				
*Recorded co-pathologies*				
Age-related macular degeneration	11 055 (10.5)	5265 (9.3)	1706 (9.2)	18 026 (10.0)
Glaucoma	9105 (8.7)	4155 (7.4)	1150 (6.2)	14 410 (8.0)
Diabetic retinopathy	5018 (4.8)	2615 (4.6)	832 (4.5)	8465 (4.7)
Other	5068 (4.8)	1536 (2.7)	436 (2.4)	7040 (3.9)
High myopia	4354 (4.1)	1657 (2.9)	546 (2.9)	6557 (3.6)
Brunescent/white cataract	3616 (3.4)	1272 (2.3)	298 (1.6)	5186 (2.9)
Corneal pathology	2806 (2.7)	1173 (2.1)	267 (1.4)	4246 (2.4)
Previous vitrectomy surgery	1830 (1.7)	699 (1.2)	389 (2.1)	2918 (1.6)
Amblyopia	1612 (1.5)	788 (1.4)	280 (1.5)	2680 (1.5)
Pseudoexfoliation/phacodonesis	1706 (1.6)	422 (0.7)	99 (0.5)	2227 (1.2)
Uveitis/synaechiae	1376 (1.3)	305 (0.5)	89 (0.5)	1770 (1.0)
Other macular pathology	939 (0.9)	453 (0.8)	155 (0.8)	1547 (0.9)
Other retinal vascular pathology	886 (0.8)	479 (0.8)	155 (0.8)	1520 (0.8)
No fundal view/vitreous opacities	923 (0.9)	359 (0.6)	79 (0.4)	1361 (0.8)
Optic nerve/CNS disease	436 (0.4)	213 (0.4)	56 (0.3)	705 (0.4)
Inherited eye diseases	160 (0.2)	51 (0.1)	15 (0.1)	226 (0.1)

**Table 2:** Intraoperative complications in the operated eye by grade of operating surgeon

*Intraoperative complications,* n *(column %)*	*Consultants (*n=*105* *116)*	*Independent non-consultants (*n=*56* *359)*	*Trainee (*n=*18* *639)*	*Total (*n=*180* *114)*
No intraoperative complication	101 392 (96.5)	53 762 (95.4)	17 460 (93.7)	172 614 (95.8)
One or more intraoperative complication	3724 (3.5)	2597 (4.6)	1179 (6.3)	7500 (4.2)
				
*Reported intraoperative complications*				
Posterior capsule rupture and/or vitreous loss (PCR)	1701 (1.6)	1222 (2.2)	591 (3.2)	3514 (2.0)
Other	658 (0.6)	412 (0.7)	148 (0.8)	1218 (0.7)
Iris trauma/prolapse	416 (0.4)	330 (0.6)	155 (0.8)	901 (0.5)
Zonule dialysis	477 (0.5)	277 (0.5)	116 (0.6)	870 (0.5)
Corneal epithelial abrasion	237 (0.2)	190 (0.3)	73 (0.4)	500 (0.3)
Endothelial damage/descemet's tear	187 (0.2)	112 (0.2)	105 (0.6)	404 (0.2)
Nuclear/epinuclear fragment into vitreous*	143 (0.1)	120 (0.2)	53 (0.3)	316 (0.2)
Corneal oedema	106 (0.1)	98 (0.2)	50 (0.3)	254 (0.1)
Lens exchange required/other IOL problems	111 (0.1)	82 (0.1)	19 (0.1)	212 (0.1)
Phaco burn/wound problems	83 (<0.1)	47 (<0.1)	21 (0.1)	151 (<0.1)
Hyphaema	60 (<0.1)	27 (<0.1)	12 (<0.1)	99 (<0.1)
Choroidal/suprachoroidal haemorrhage	53 (<0.1)	27 (<0.1)	9 (<0.1)	89 (<0.1)

* this complication is reported separately and as part of the PCR results.

**Table 3:** Postoperative visual acuities

*Variable*	*Number*	*≤0.00 (6/6)*	*≤0.18 (6/9)*	*≤0.30 (6/12)*	*≤0.60 (6/24)*
*Percentage of eyes with best-measured postoperative visual acuity equal to or better than, logMAR (Snellen approximations)*					
All eyes	95 561	44.5	53.2	89.6	95.9
Eyes with ocular co-pathology	32 713	32.5	41.1	79.9	90.7
Eyes without ocular co-pathology	62 848	50.8	59.5	94.6	98.6
Eyes with an intraoperative complication	3153	31.2	38.6	80.6	90.2
Eyes with no intraoperative complications	92 408	45.0	53.7	89.9	96.1
Eyes with PCR	1342	27.9	36.0	76.8	87.0
Eyes with no PCR	94 219	44.7	53.5	89.7	96.0
					
*Percentage of eyes with postoperative UDVA visual acuity equal to or better than, logMAR (Snellen approximations) and a predictive postoperative refraction of* ±*0.5 dioptres*					
All eyes	63 669	23.8	29.7	75.3	93.2
Eyes with ocular co-pathology	20 092	16.2	21.2	63.2	86.2
Eyes without ocular co-pathology	43 577	27.3	33.6	80.9	96.4
Eyes with an intraoperative complication	1935	14.2	18.8	60.6	84.6
Eyes with no intraoperative complications	61 734	24.1	30.0	75.7	93.4
Eyes with PCR	777	10.8	15.2	54.8	79.9
Eyes with no PCR	62 892	24.0	29.9	75.5	93.3

**Table 4:** Visual loss

*Visual loss, n (column %)*	*Baseline visual acuity equal to or better than, logMAR (Snellen approximations)*				*Overall*
	*≤0.00 (6/6)*	*≤0.18 (6/9)*	*≤0.30 (6/12)*	*≤0.60 (6/24)*	
*All eyes*					
Number	3058	4197	17 485	57 798	94 106
Visual loss	210 (6.9)	234 (5.6)	507 (2.9)	1000 (1.7)	1455 (1.5)
					
*Eyes with no ocular co-pathology*					
Number	2277	3131	12 927	41 083	62 848
Visual loss	130 (5.7)	144 (4.6)	316 (2.4)	544 (1.3)	650 (1.0)
					
*Eyes with an ocular co-pathology*					
Number	781	1066	4558	16 715	32 713
Visual loss	80 (10.2)	90 (8.4)	191 (4.2)	456 (2.7)	805 (2.5)
					
*Eyes without an operative complication*					
Number	2982	4098	17 018	56 134	92 408
Visual loss	197 (6.6)	220 (5.4)	471 (2.8)	900 (1.6)	1305 (1.4)
					
*Eyes with an operative complication*					
Number	76	99	467	1664	3153
Visual loss	13 (17.1)	14 (14.1)	36 (7.7)	100 (6.0)	150 (4.8)
					
*Eyes with no PCR*					
Number	3029	4155	17 279	57 117	94 219
Visual loss	201 (6.6)	224 (5.4)	481 (2.8)	935 (1.6)	1357 (1.4)
					
*Eyes with PCR*					
Number	29	42	206	681	1342
Visual loss	9 (31.0)	10 (23.8)	26 (12.6)	65 (9.5)	98 (7.3)
